# Fabrication of Nanosized Island-Like CdO Crystallites-Decorated TiO_2_ Rod Nanocomposites via a Combinational Methodology and Their Low-Concentration NO_2_ Gas-Sensing Behavior

**DOI:** 10.3390/ma10070778

**Published:** 2017-07-10

**Authors:** Yuan-Chang Liang, Nian-Cih Xu, Chein-Chung Wang, Da-Hua Wei

**Affiliations:** 1 Institute of Materials Engineering, National Taiwan Ocean University, Keelung 20224, Taiwan; sad821008@gmail.com (N.-C.X.); abc2589tw@gmail.com (C.-C.W.); 2 Graduate Institute of Manufacturing Technology and Department of Mechanical Engineering, National Taipei University of Technology, Taipei 10608, Taiwan

**Keywords:** sputtering, microstructure, rod nanocomposite, sensing performance

## Abstract

TiO_2_–CdO composite rods were synthesized through a hydrothermal method and sputtering thin-film deposition. The hydrothermally derived TiO_2_ rods exhibited a rectangular cross-sectional crystal feature with a smooth surface, and the as-synthesized CdO thin film exhibited a rounded granular surface feature. Structural analyses revealed that the CdO thin film sputtered onto the surfaces of the TiO_2_ rods formed a discontinuous shell layer comprising many island-like CdO crystallites. The TiO_2_–CdO composite rods were highly crystalline, and their surfaces were rugged. A comparison of the NO_2_ gas-sensing properties of the CdO thin film, TiO_2_ rods, and TiO_2_–CdO composite rods revealed that the composite rods exhibited superior gas-sensing responses to NO_2_ gas than did the CdO thin film and TiO_2_ rods, which can be attributed to the microstructural differences and the formation of heterojunctions between the TiO_2_ core and CdO crystallites.

## 1. Introduction

Gas sensors based on oxide semiconductors, such as TiO_2_, have the potential to detect harmful and toxic gases with high sensitivity [1,2,3,4]. TiO_2_ nanostructures have a large surface-to-volume ratio because of their one-dimensional structure, which is advantageous in providing high surface effects between the oxide surface and the detected gases. Several studies have investigated methods to fabricate TiO_2_-based one-dimensional structures with various morphologies for application as gas-sensing devices. For example, the high acetone gas-sensing response of electrospinning-synthesized TiO_2_ nanorods was investigated at 500 °C [5]. TiO_2_ nanotubes synthesized through anodization of Ti foil at room temperature were used to detect H_2_ gas [6]. TiO_2_ nanowires were synthesized by subjecting sol–gel derived solid thin films to a photolithographic process for application in visible ethanol vapor sensing [7]. TiO_2_ nanofibers are formed via the reaction of dense polycrystalline TiO_2_ in a H_2_/N_2_ environment at an elevated temperature and are investigated as gas sensing materials [8]. Moreover, gas sensors made from nano-heterostructures are shown to be a promising approach to enhance the gas-sensing response of the oxide semiconductors [9]. To further improve the gas-sensing performance of sensors composed of pure TiO_2_ nanostructures, TiO_2_ nanostructures on the surfaces of these sensors can be modified by using binary oxides such as ZnO, SnO_2_, and In_2_O_3_. Many core–shell structures of TiO_2_-based nanomaterials have been synthesized, and their applications in gas sensing have been investigated. ZnO sheet-decorated TiO_2_ fibers, synthesized through a combination of the electrospinning technique and the hydrothermal growth method, exhibited a response of 15.7 to 100 ppm ethanol at 280 °C, which is much higher than that of pure TiO_2_ and ZnO [10]. Heterostructures comprising SnO_2_ nanoparticles deposited on TiO_2_ nanobelts through a hydrothermal method exhibited high gas-sensing sensitivity for acetone vapor because of their large surface area [11]. Porous In_2_O_3_/TiO_2_ composite nanofibers synthesized through a facile electrospinning-based synthesis method, followed by appropriate thermal treatment under ambient conditions, exhibited higher gas-sensing responses to NO_x_ gas than those of pure TiO_2_ nanofibers [12].

Few studies have used CdO—another promising gas-sensing binary oxide semiconductor—to decorate the surfaces of TiO_2_ for improving its gas-sensing performance toward various toxic gases. CdO has a bandgap in the visible-light region, and its native oxygen vacancies make it an n-type semiconductor with low resistance [13]. CdO thin films and nanostructures have been synthesized and examined through various physical and chemical techniques [14,15,16]. For example, the gas-sensing properties of chemical bath-deposited CdO thin films were studied for sensing liquefied petroleum gas at an operating temperature of 300 °C [17]. Highly crystalline CdO nanostructures prepared through microwave-assisted growth were investigated as the sensing layer in resistive sensors and tested for NO_2_ sensing [18]. Recently, gas sensors fabricated using composite materials incorporating CdO have been shown to exhibit superior gas-sensing performance than do the constituent individual oxides. For example, two-phase mixed CdO–MnO_2_ thin films enhanced the performance of the constituent individual oxide films when sensing reduced gases [19]. Moreover, the formation of CdO–ZnO heterostructures improved the applicability of CdO in gas sensors [20]. Incorporating CdO into one-dimensional TiO_2_ structures is promising for improving the gas-sensing response of one-dimensional TiO_2_-based sensors. However, limited studies have investigated the gas-sensing properties of one-dimensional TiO_2_–CdO composites. Therefore, in this study, island-like CdO crystallites were decorated onto TiO_2_ rods to form a core–shell structure, and the correlation between the microstructure and the gas-sensing performance of TiO_2_–CdO composite rods for sensing low-concentration NO_2_ gas was investigated.

## 2. Materials and Methods

In this study, TiO_2_ rods sputtering deposited a 100-nm-thick CdO thin film and were used as samples for investigating the structure-dependent NO_2_ gas-sensing response of the novel TiO_2_–CdO composite rods. The TiO_2_ rods were grown on glass substrates using a hydrothermal method in this study. An amount of 7.4 mL of DI water was mixed with 12.6 mL of concentrated HCl (35%) in a Teflon-lined digestion autoclave. The mixture was stirred at ambient conditions for 5 min and after that, 0.25 mL of TiCl_4_ was added into the mixed solution for preparation of the TiO_2_ rods. The hydrothermal synthesis was conducted at 180 °C for 3 h. After the synthesis reaction, the autoclave was cooled down to room temperature. Then the samples were removed, washed with DI water, and air dried. TiO_2_–CdO composite rods were fabricated by sputtering CdO thin films onto the surfaces of the hydrothermally derived TiO_2_ rod templates. The CdO thin films were fabricated through RF magnetron sputtering using a CdO target in mixed Ar/O_2_ ambient with a ratio of 20/5 at 350 °C. The working pressure during thin-film deposition was fixed at 2.67 Pa, and the RF sputtering power was fixed at 60 W.

Crystal structures of the as-synthesized samples were investigated by X-ray diffraction (XRD; D2 PHASER, Bruker, Karlsruhe, Germany) using Cu Kα radiation. The surface morphologies of the samples were characterized by scanning electron microscopy (SEM; S-4800, Hitachi, Tokyo, Japan). High-resolution transmission electron microscopy (HRTEM; Tecnai F20 G2, Philips, Amsterdam, The Netherlands) was used to investigate the detailed microstructures of the TiO_2_–CdO composite rods. The attached energy dispersive X-ray spectroscopy (EDS) was used to investigate the composition of the TiO_2_ and TiO_2_–CdO rod samples. The analyses of the transmittance and reflectance spectra of various samples were conducted by using a UV-Vis spectrophotometer (V750, Jasco, Tokyo, Japan). An X-ray photoelectron spectroscopy (XPS; ULVAC-PHI XPS, ULVAC, Chigasaki, Japan) analysis was used to determine the chemical binding status of the constituent elements of the samples. The gas sensing system used in this study consists of a gas flow system consisting of two lines. One is for a cylinder containing the background gas N_2_; the other one was connected to a cylinder containing diluted NO_2_ gas. The NO_2_ concentration for the gas-sensing tests was varied from 1.0 ppm to 5.0 ppm by controlling the relative flow rates of background gas and target gas through mass flow controllers. Before conducting the gas-sensing tests, the test chamber was pumped to a vacuum environment. Subsequently, highly pure nitrogen gas at a consistent flow rate of 1000 sccm was introduced into the test chamber for 10 min; thereafter, the sample holder was heated to the desired temperatures using a direct heating approach. After the electric resistance of the sensors was stabilized, NO_2_ gas at concentrations of 1.0, 2.5, and 5.0 ppm were introduced into the test chamber to investigate the NO_2_ gas-sensing performance of the samples. The gas-sensing response of the sensors made from the CdO film, TiO_2_ rods and TiO_2_–CdO rods to NO_2_ gas is defined as the Rg/Ra. Ra is the sensor electrical resistance in the absence of a target gas, and Rg is that in the target gas. The measurements of a gas-sensing response were conducted with various concentrations of NO_2_ gas. The 100 ppm CO, H_2_, and NH_3_ gases were used for a gas-sensing selectivity test. The gas-sensing responses for these reducing gases are defined as Ra/Rg.

## 3. Result and Discussion

**Figure 1:**
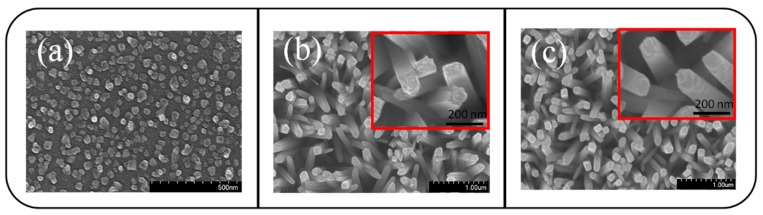
(**a**) SEM image of the CdO film. (**b**) SEM image of the TiO_2_ rods. (**c**) SEM image of the TiO_2_–CdO rods.

Figure 1a shows the surface topography of a CdO film. The surface was rough and comprised many rounded, surface granular crystallites with a diameter of 20–45 nm. Figure 1b,c depict the morphologies of the TiO_2_ and TiO_2_–CdO composite rods. The TiO_2_ rods were dense and homogeneously distributed over the substrate. The pure TiO_2_ rods exhibited a smooth surface (inset, Figure 1b). The diameter of the TiO_2_ rods ranged from 100 to 140 nm, and the tip of the TiO_2_ rods exhibited a rectangular feature. By contrast, the TiO_2_ rods coated with CdO shell layers exhibited a rugged surface feature and high surface roughness (Figure 1c). High-magnification SEM revealed that the surfaces of the TiO_2_–CdO composite rods were comprised of many small, irregular-shaped, and clustered crystallites (inset, Figure 1c).

**Figure 2:**
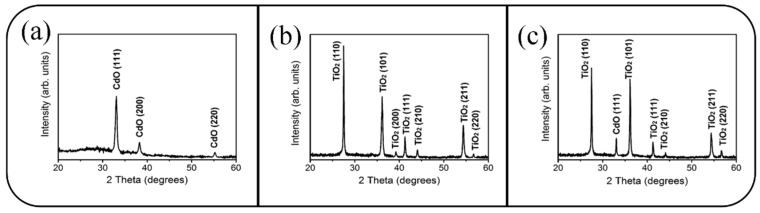
(**a**) XRD pattern of the CdO film. (**b**) XRD pattern of the TiO_2_ rods. (**c**) XRD pattern of the TiO_2_–CdO rods.

Figure 2a shows the XRD pattern of a 100-nm-thick CdO thin film. The three distinct Bragg reflections centered at approximately 33.02°, 38.3°, and 55.3° can be ascribed to the (111), (200), and (220) planes of cubic-phase CdO (JCPDS 005-0640). The multiple Bragg reflections in the XRD pattern revealed that the as-synthesized CdO thin film has a polycrystalline feature. Notably, the intense Bragg reflection of (111) revealed that most CdO grains in the film were highly oriented (111). The XRD patterns of the as-synthesized TiO_2_ rods (Figure 2b) revealed several marked Bragg reflections centered at approximately 27.5°, 36.0°, 39.3°, 41.1°, 44.1°, 54.2°, and 56.7°, which can be ascribed to the crystallographic plane of rutile TiO_2_ (JCPDS 001-1292); this result indicated that the as-synthesized TiO_2_ rods are polycrystalline. Figure 2c presents the XRD pattern of the TiO_2_ rods decorated with CdO crystallites through sputtering deposition, in which the Bragg reflection centered at approximately 33.02° can be ascribed to cubic CdO(111); the appearance of this reflection was consistent with the XRD findings for the CdO thin film, which indicated that the sputtering-deposited CdO thin film had a highly (111)-oriented crystallographic feature.

**Figure 3:**
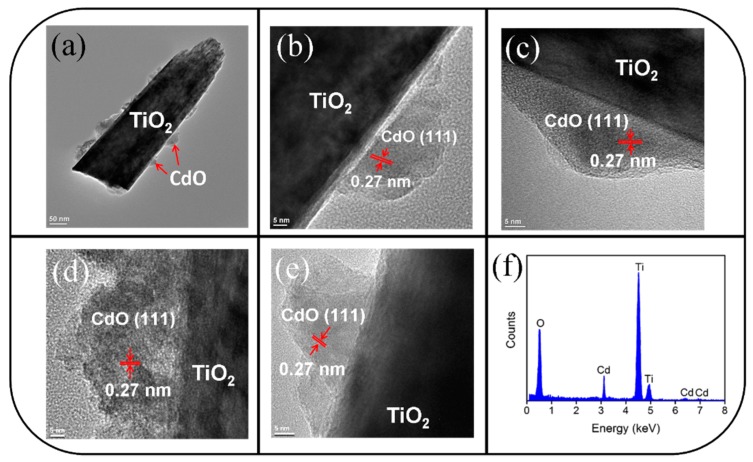
TEM analyses of the TiO_2_–CdO composite rod: (**a**) Low-magnification TEM image of the TiO_2_–CdO rod; (**b**–**e**) HRTEM images taken from the local regions of the rod; (**f**) EDS spectra of Ti, Cd, and O elements taken from the rod.

Figure 3a is a low-magnification TEM image of a single TiO_2_–CdO composite rod. As shown in the figure, the surface morphology of the shell layer indicated that this layer was comprised of many discontinuous island-like crystallites, and TiO_2_–CdO exhibited a rugged surface feature. The thickness distribution of the island-like CdO shell layer over the CdO crystallites was inhomogeneous and ranged from 15 to 30 nm. Notably, the thickness of the CdO shell layer on the TiO_2_ rod surface was less than the original two-dimensional 100-nm-thick CdO film; this was because the deposition of the two-dimensional continuous CdO film onto the three-dimensional free-standing TiO_2_ rods engendered a large surface dispersion effect and decreased the coverage thickness of the CdO on the TiO_2_ surface [21]. Figure 3b–e show the high-resolution TEM images of the various outer regions of the composite rod. Several boundaries in the CdS crystallites were visible in these images. The clear and ordered lattice fringes with an interval of approximately 0.27 nm in the CdO crystal corresponded to the {111} lattice plane. The crystalline CdO phase was sputtered onto the surface of the TiO_2_ rod. EDS spectroscopy revealed that Ti, Cd, and O are the main constituent elements of the composite rod (Figure 3f).

**Figure 4:**
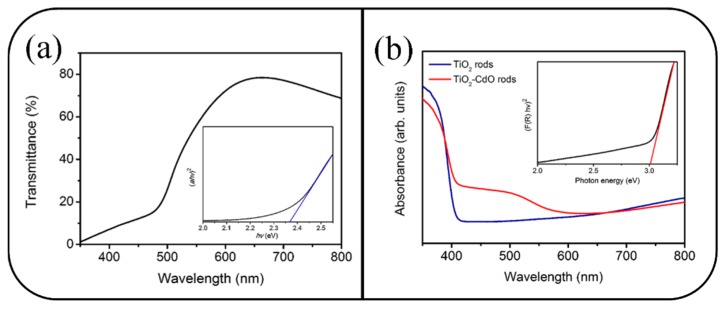
(**a**) Transmittance spectrum of the CdO film. The inset in (**a**) shows the Tauc plot of the CdO film. (**b**) Absorbance spectra of the TiO_2_ and TiO_2_–CdO rods. The inset in (**b**) shows the bandgap of the TiO_2_ rods is approximately 3.02 eV.

Figure 4a shows the optical transmittance spectrum of the CdO film. The transmittance of the film substantially decreased in the wavelength range of 500–600 nm, indicating that the CdO film has a bandgap in the visible-light region. The optical bandgap of the CdO film was evaluated using the Tauc model [22]. The inset in Figure 4a illustrates the plot of (*ahv*)^2^ vs. *hv*, where *a* is the absorption coefficient and *hv* is the photon energy. The bandgap of the CdO film, calculated by extrapolating the linear portion of the curve until it intercepted the horizontal axis (photon energy), was approximately 2.36 eV. Furthermore, the diffuse reflectance spectra of the TiO_2_ and TiO_2_–CdO rods were recorded and converted into absorption coefficient spectra by applying the Kubelka–Munk function [23] (Figure 4b). The optical absorbance edge of the TiO_2_ rods was located in the UV region at a wavelength of approximately 400 nm. The optical bandgap of the TiO_2_ rods was 3.02 eV (the inset in Figure 4b), which is similar to that reported for rutile TiO_2_ [24]. Notably, the optical absorbance edge of the TiO_2_–CdO rods broadened and extended to the visible-light region. This clear red-shift of the optical absorbance edge of the TiO_2_–CdO rods demonstrated the successful decoration of the CdO crystallites, which has a narrower bandgap, onto the surfaces of the TiO_2_ rods through sputtering.

**Figure 5:**
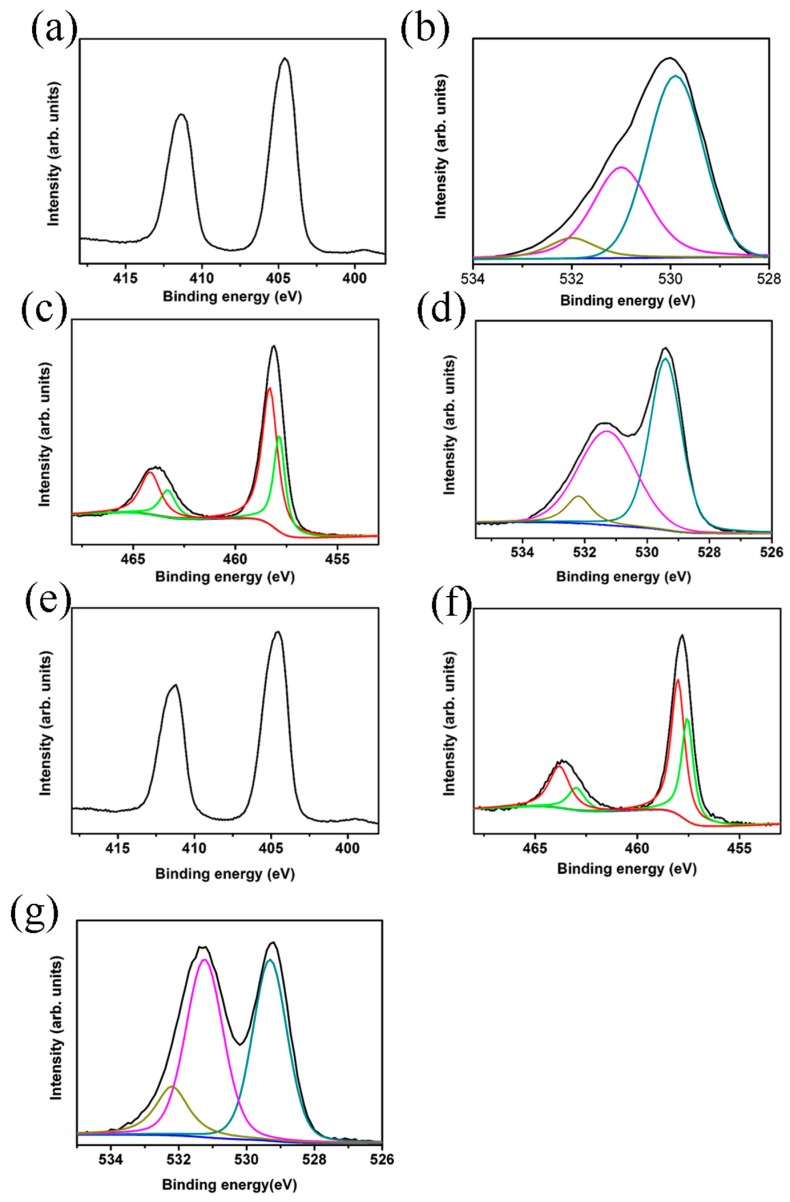
XPS narrow scan analyses for the CdO thin film, TiO_2_ rods, and TiO_2_–CdO composite rods: (**a**) Cd 3d core-level doublet peaks of the CdO thin film; (**b**) O1s core-level spectrum of the CdO thin film; (**c**) Ti 2p core-level spectra of the TiO_2_ rods; (**d**) O1s core-level spectrum of the TiO_2_ rods; (**e**) Cd 3d core-level doublet peaks of the TiO_2_–CdO composite rods; (**f**) Ti 2p core-level spectra of the TiO_2_–CdO composite rods; (**g**) O1s core-level spectrum of the TiO_2_–CdO composite rods.

Figure 5a presents the narrow-scan Cd 3d XPS spectrum for the CdO film. The Cd 3d XPS spectrum contained the main 3d_5/2_ and 3d_3/2_ spin-orbit components with the binding energies of approximately 404.56 and 411.36 eV, respectively. These binding energies are ascribed to the Cd^2+^ bonding state in the CdO phase [25]. Figure 5b shows the O1s spectrum of the CdO film. The asymmetricity of the spectrum revealed that the as-synthesized CdO film contained oxygen in multiple valence states. The low-binding-energy peak can be attributed to the Cd–O bonds, the medium-binding-energy peak results from the oxygen vacancy in the oxide lattice, and the relatively high-binding-energy peak is associated with the chemisorbed oxygen species on the film surface [26]. Figure 5c depicts the Ti 2p core spectra of the TiO_2_ rods. The 2p_3/2_ and 2p_1/2_ peaks were deconvoluted into four subpeaks. The subpeaks located in the higher-binding-energy regions of the 2p_3/2_ and 2p_1/2_ peaks can be assigned to the Ti^4+^ valence state, whereas those located in the lower-binding-energy regions can be assigned to the Ti^3+^ valance state. The presence of the mixed Ti^4+^/Ti^3+^ valance state indicated the presence of oxygen vacancies in the surfaces of the as-synthesized TiO_2_ rods [27]. The O1s spectrum of the TiO_2_ rods exhibited a marked asymmetric curve feature (Figure 5d). The O1s peak was deconvoluted into three subpeaks. The low-binding-energy component can be attributed to the Ti–O bonds, the medium-binding-energy component results from the oxygen vacancy in the oxide lattice, and the high-binding-energy component can be ascribed to the oxygen species chemisorbed from the ambient air [28]. The integrated area ratio of chemisorbed oxygen and the oxygen vacancy was 4.3% and approximately 43.8%, respectively. Figure 5e shows the XPS spectral signal of Cd 3d associated with the island-like CdO crystallites decorated onto the surfaces of the TiO_2_ rods. Figure 5f shows the Ti 2p core spectra of the surfaces of the TiO_2_ rods in the TiO_2_–CdO core–shell composites, which clearly demonstrates the mixed Ti^4+^/Ti^3+^ valance state in the TiO_2_ rods. No substantial differences were observed in the Ti^4+^/Ti^3+^ valance state of TiO_2_ rods with and without decoration with the island-like CdO crystallites. By contrast, the O1s spectral intensity substantially increased in the relatively high-binding-energy region of the TiO_2_ rods after decoration, revealing an increase in the number of crystal defects and chemisorbed oxygen species on the surfaces of the composite rods [2]. In addition, when the TiO_2_ rods were decorated with the island-like CdO crystallites, the integrated area ratio of chemisorbed oxygen and oxygen vacancy increased to 8.4% and 45.5%, respectively (Figure 5g). The increased oxygen vacancy in the composite rods can be attributed to the intrinsic crystal defects in the CdO crystallites, and the increase in the number of chemisorbed oxygen species can be attributed to the increase in the surface area of the composite rods due to its rugged surface morphology.

**Figure 6:**
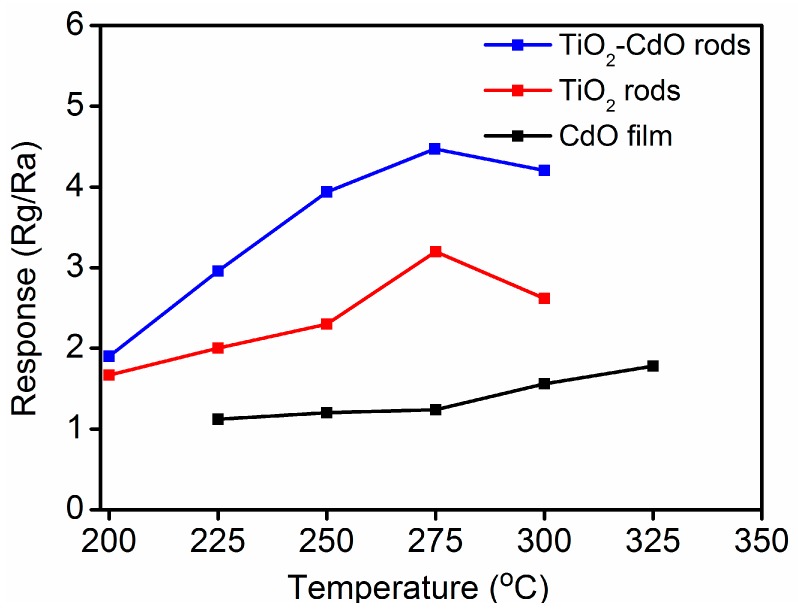
Gas sensing response value vs. operating temperature of the CdO film, TiO_2_ rods, and TiO_2_–CdO rods.

Figure 6 presents the gas-sensing responses of the CdO thin film, TiO_2_ rods, and TiO_2_–CdO composite rods as a function of the operating temperature on exposure to 1.0 ppm NO_2_ gas. To prevent microstructural changes in the deposited CdO thin film, the operating temperature was not increased beyond the CdO thin-film growth temperature of 350 °C. The CdO thin film exhibited its maximum gas-sensing response at a high operating temperature of 325 °C. By contrast, the gas-sensing response of the TiO_2_ rods and the TiO_2_–CdO rods peaked at 275 °C. The optimal operating temperature of the oxide sensors is highly associated with the balance between the chemical reactions and the gas diffusion rate [1,29]. Therefore, the differences in the specific surface area of the samples might crucially affect the optimal operating temperatures of the investigated samples. For example, because of its relatively low specific surface area, the CdO thin film required a relatively higher operating temperature to achieve effective chemisorption between the film surface and the reaction gas. By contrast, in one-dimensional TiO_2_ rods—which have a higher specific surface area than do the two-dimensional CdO thin films—equilibrium between the surface reaction with NO_2_ gas and the diffusion of NO_2_ gas to the surface of the TiO_2_ rods was achieved at a relatively low sensor operating temperature of 275 °C. The optimal operating temperature of the TiO_2_–CdO rods was similar to that of the TiO_2_ rods, revealing that the decoration of the TiO_2_ rods with island-like CdO crystallites did not markedly alter the temperature at which the aforementioned equilibrium was achieved.

**Figure 7:**
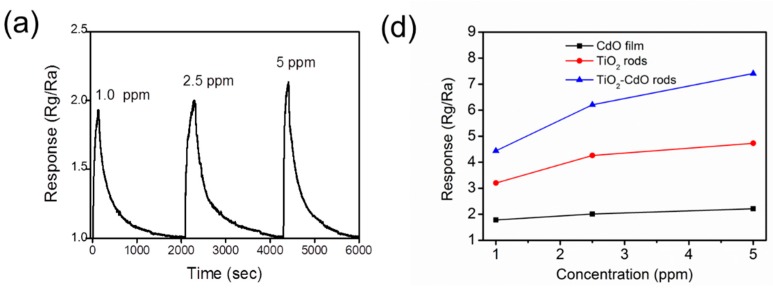

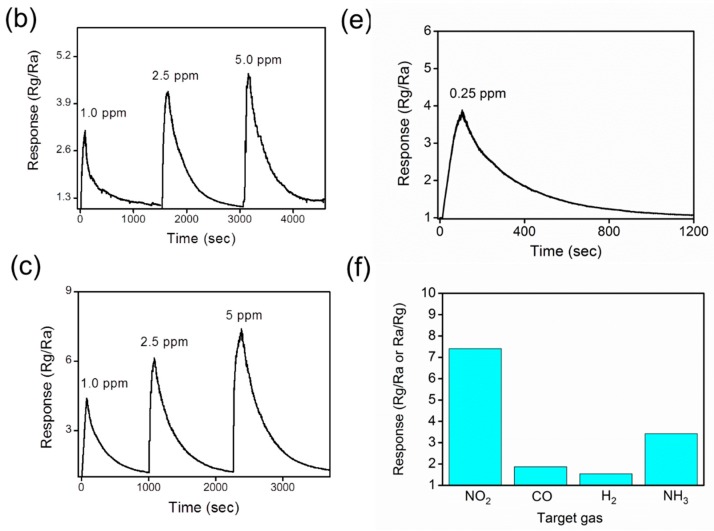
Gas sensing response curves of various samples on exposure to various concentrations of NO_2_ gas (1.0, 2.5, and 5.0 ppm) at various temperatures: (**a**) CdO film at 325 °C; (**b**) TiO_2_ rods at 275 °C; (**c**) TiO_2_–CdO rods at 275 °C; (**d**) Gas sensing response values vs. NO_2_ gas concentration for various samples; (**e**) Gas sensing response curve of the TiO_2_–CdO rods on exposure to 0.25 ppm NO_2_ gas; (**f**) Gas sensing selectivity histogram of the TiO_2_–CdO rods tested to 5 ppm NO_2_ and 100 ppm of CO, H_2_, and NH_3_.

Figure 7a–c show the gas-sensing response curves of the CdO film, TiO_2_ rods, and TiO_2_–CdO rods on exposure to various NO_2_ gas concentrations, respectively. The sensor operating temperature for the CdO film is 325 °C and those for the TiO_2_ and TiO_2_–CdO rods are 275 °C. TiO_2_ and CdO are n-type semiconductors; therefore, the resistance of the fabricated sensors increased upon their exposure to NO_2_ gas. This is because the adsorption of NO_2_ gas molecules onto the surfaces of the CdO film, TiO_2_ rods, and TiO_2_–CdO rods engendered the extraction of surface electrons, resulting in an increase in sensor resistance [30]. The reactions between the NO_2_ gas molecules and the surface electrons of the CdO film, TiO_2_ rods, and TiO_2_–CdO rods are as follows [30]:

(1) NO_2_(g) + e^−^ → NO_2_^−^(ads)

(2) NO_2_(g)+ e^−^ → NO(g) + O^−^(ads)

The CdO film, TiO_2_ rods, and TiO_2_–CdO rods exhibited evident NO_2_ gas-sensing behavior on exposure to various NO_2_ gas concentrations (Figure 7a–c); their gas-sensing responses are summarized in Figure 7d. The responses of the CdO film to 1.0, 2.5, and 5.0 ppm NO_2_ were approximately 1.78, 2.01, and 2.21, respectively. Those of the TiO_2_ rods were approximately 3.21, 4.26, and 4.73, respectively, and those of the TiO_2_ rods decorated with CdO crystallites were higher, at 4.44, 6.21, and 7.41, respectively. The gas-sensing responses of the TiO_2_−CdO rods were higher than those of the TiO_2_ rods and CdO film at all investigated NO_2_ gas concentrations, whereas the CdO film exhibited the lowest responses. In general, the gas-sensing responses of the CdO film, TiO_2_ rods, and TiO_2_–CdO rods increased with the NO_2_ concentration because of the increase in the number of NO_2_ gas molecules interacting with the surfaces of the samples in the NO_2_-rich test environment. However, the gas-sensing responses of the CdO film increased by only approximately 24% as the NO_2_ gas concentration increased from 1.0 to 5.0 ppm, whereas the corresponding change for the TiO_2_ rods and TiO_2_–CdO rods was 48% and 67%, respectively. Clearly, the one-dimensional TiO_2_ rods with a relative large specific surface area are more sensitive to NO_2_ gas at various gas concentrations than is the CdO film, whereas the sensitivity of TiO_2_–CdO rods to NO_2_ gas is even higher than that of the TiO_2_ rods. This result can be attributed to the differences in the rod surface morphology and the formation of heterojunctions between the TiO_2_ core and the CdO shell. The rugged and discontinuous island-like CdO crystallites on the surfaces of the TiO_2_ rods provided more sites for NO_2_ gas adsorption, as has been reported for one-dimensional semiconductors [31,32]. In addition, crystalline defects, such as surface oxygen vacancies and numerous boundaries in the island-like CdO crystallites, are favorable sites for NO_2_ absorption, and the presence of more such defects on the surfaces of the TiO_2_–CdO rods than on the surfaces of the TiO_2_ rods improved the adsorption efficiency of the TiO_2_ rods decorated with island-like CdO crystallites. In particular, an increase in oxygen vacancy defects in oxides is highly correlated with an increase in their gas-sensing performance [33]. A similar enhancement in gas-adsorption efficiency has been achieved for TiO_2_ nanobelts and ZnO nanorods through surface decoration of SnO_2_ and TiO_2_ nanoparticles, respectively [11,34]. The formation of TiO_2_–CdO core–shell heterostructures is expected to bend the energy bands at the TiO_2_/CdO interface. The interface potential barrier might be another factor to dominate the superior NO_2_ gas-sensing sensitivity of the TiO_2_–CdO rods than that of the pure TiO_2_ rods. Notably, the gas-sensing sensitivity of the TiO_2_–CdO rods on exposure to 0.25 ppm NO_2_ gas was still visible and reached to 3.91 in Figure 7e; however, no reliable sensing response curve was obtained for the pure TiO_2_ rods in this work. Furthermore, the gas-sensing selectivity of the TiO_2_–CdO rods was investigated on exposure to various target gases in Figure 7f. Comparatively, the TiO_2_–CdO rods are more sensitive to detect low-concentration NO_2_ gas in this work. The response times of the TiO_2_–CdO rods are approximately 41–53 s on exposure to 1.0–5.0 ppm NO_2_ gas, respectively. The recovery times are in the range of 305–341 s when the TiO_2_-CdO rods were exposed to 1.0–5.0 ppm NO_2_ gas, respectively. Table 1 summarized NO_2_ gas-sensing performance of other one-dimensional TiO_2_-based heterostructures prepared by various methods [35,36,37,38]. Comparatively, the TiO_2_–CdO rods have potential in gas sensor applications in detecting low-concentration NO_2_ gas.

**Figure 8:**
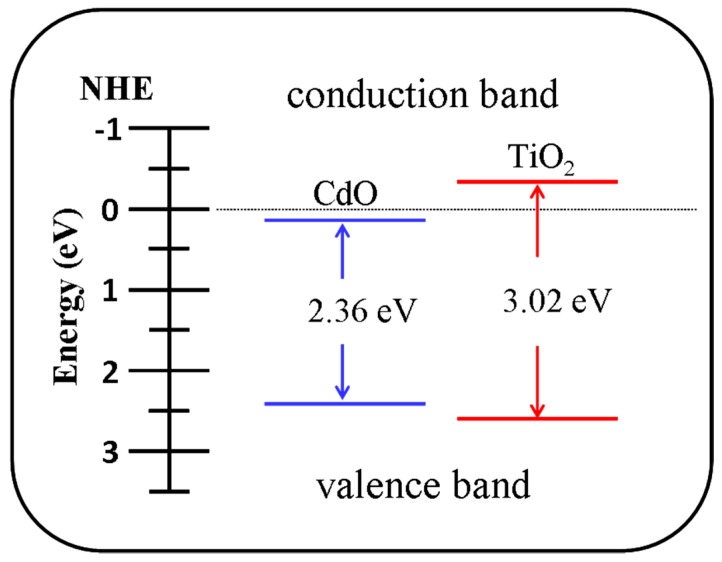
Energy diagram of the TiO_2_ and CdO phases.

**Figure 9:**
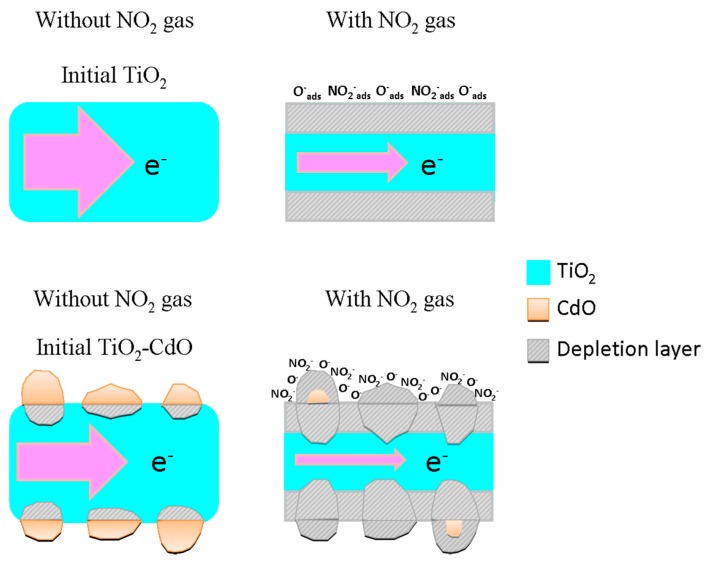
A schematic of gas sensing mechanisms for the TiO_2_ and TiO_2_–CdO rods on exposure to NO_2_ gas. The size of arrows represented the current degree in the TiO_2_.

By contrast, a formation of the heterojunction between the TiO_2_ and CdO is also an important factor that affects the NO_2_ gas-sensing response of the composite rods. Figure 8 depicts the energy diagram for the TiO_2_ and CdO phases [39,40]. As evident from the figure, many discontinuous electron-depleted regions were formed at the TiO_2_ core side when the island-like CdO crystallites discontinuously covered the surfaces of the TiO_2_ rods. Figure 9 illustrates the effect of interfacial potential barriers on the NO_2_ gas-sensing response of the TiO_2_ rods decorated with CdO crystallites. Many discontinuous depletion regions were initially formed over the surface of the TiO_2_ rods after they were coated with the CdO crystallites. Upon exposure to NO_2_ gas, the adsorption of the NO_2_ gas molecules onto the surface of the TiO_2_ rods resulted in the extraction of the surface electrons. This further induced the formation of a layer-like surface-depletion region on the TiO_2_ rod surface. The presence of this surface-depletion region markedly decreased the channel size for carrier transportation, increasing the resistance of the sensor made from the TiO_2_ rods. By contrast, when the TiO_2_–CdO rod was exposed to the NO_2_ gas, the inhomogeneous coverage of the island-like CdO crystallites absorbed more NO_2_ gas molecules onto the surface of the TiO_2_–CdO rod than did the pure TiO_2_ rod; this is because of the defective crystalline feature of the CdO and the increased surface area of the composite rod. The relatively large fluctuation in the charge density, caused by the adsorption of NO_2_ gas molecules onto the surfaces of the TiO_2_–CdO rods, further affected the size variation of the depletion region [31]. Consequently, the thickness distribution of the depletion regions over the TiO_2_ rod surface became more inhomogeneous, substantially decreasing the carrier transportation efficiency in the TiO_2_ rods. Therefore, the variation in sensor resistance on exposure to NO_2_ gas would be substantially larger for the TiO_2_–CdO rods than for the pure TiO_2_ rods. On the basis of the microstructural differences between the TiO_2_ and TiO_2_–CdO rods and the formation of numerous interfacial depletion regions in the TiO_2_–CdO rod, it can be concluded that the TiO_2_–CdO rods exhibit the highest NO_2_ gas detection ability among the samples investigated in this study.

## 4. Conclusions

In summary, TiO_2_–CdO core–shell composite rods were initially synthesized through a combination of hydrothermal and sputtering methods. Electron microscopy analyses revealed that the surfaces of the as-synthesized TiO_2_ rods were smooth. Moreover, the decoration of CdO thin film onto the surfaces of the TiO_2_ rods engendered the formation of a discontinuous shell layer, which comprised many island-like CdO crystallites of varying size. Structural analyses revealed that the as-synthesized TiO_2_–CdO composite rods were highly crystalline, and the XPS spectra provided evidence that oxygen vacancies exist in the surfaces of the TiO_2_ rods and CdO crystallites. The superior NO_2_ gas-sensing response of the TiO_2_–CdO composite rods relative to that of pure TiO_2_ rods and the CdO thin film in this study can be attributed to the increased surface area and surface defect density and the formation of the TiO_2_/CdO heterojunctions.

## Figures and Tables

**Table 1:** Comparisons of NO_2_ gas-sensing performance of various low-dimensional, TiO_2_–based composites.

Materials	Synthesis Method	NO_2_ Sensing Condition	Response	Response Time/Recovery Time	Response Definition	Ref.
TiO_2_−In_2_O_3_	Electrospinning	2.9 ppm/RT	3.5	8 s/56 s	(Rg–Ra)/Ra (N-type behavior)	[35]
TiO_2_–V_2_O_5_	Sol–gel and solvothermal methods	2 ppm/200 °C	0.8	N/A	(Rg–Ra)/Ra (N-type behavior)	[36]
TiO_2_–MoS_2_	Anodization and hydrothermal methods	100 ppm/150 °C	1.1	N/A	Ra/Rg (P-type behavior)	[37]
TiO_2_–Al_2_O_3_	Thermal oxidation	1000 ppm/650 °C	1.9	180 s/180 s	Rg/Ra (N-type behavior)	[38]
TiO_2_–CdO	Hydrothermal and sputtering methods	5 ppm/275 °C	7.41	53 s/341 s	Rg/Ra (N-type behavior)	Present work
